# Supplementary material for: Environmental non-governmental organizations and air-pollution governance: Empirical evidence from OECD countries
Source: PLoS One. 2021 Aug 12;16(8):e0255166. doi: 10.1371/journal.pone.0255166 (PMC8360548; doi:10.1371/journal.pone.0255166)
Supplement: Attachment — Submitted filename: Response to Reviewers.docx [file pone.0255166.s001.docx]

**Response to Reviewers**

First, we would like to thank reviewers for your insightful, constructive and helpful comments on our manuscript entitled “**Environmental Non-Governmental Organizations and Air-Pollution Governance: Empirical Evidence from OECD Countries**”. We have carefully considered and addressed all the comments and made necessary revisions in the revised manuscript. We provide a point-by-point response to the reviewers’ comments below.

The points raised by the reviewers are written in **bold font,** whereas our responses are shown in normal font, and the quotation of the revised manuscript is shown in *italic font*.

# Reviewer #2:

**Reviewer #2: The authors have already done remarkable work on the revision. However, I still think it is not appropriate to investigate whether ENGOs and PM2.5 have an interactive effect on residents’ health, and I recommend deleting it.**

***Response*:**

Thank you for taking time out of your busy schedule to review our paper again. I quite agree with your suggestion. In the revised draft, we delete this part of the content and modify the relevant content of the full text. I hope you will review our revised draft again. The specific revised parts are as follows:

Firstly, in the abstract, the result of residents’ life expectancy and birth rate is removed.

Secondly, in the hypothesis part, the hypothesis about life expectancy and birth rate is deleted;

Thirdly, in the description of variables and data, the description of variables and data about life expectancy and birth rate are deleted.

Fourth, in the part of mechanism analysis, we delete the empirical analysis on life expectancy and birth rate.

Fifthly, in the conclusions and policy recommendations section, we also deleted the conclusions and recommendations related to life expectancy and birth rate.
